# Does the Modality of Dental Treatment Affect the Treatment Prognosis and the Necessity of Re-Treatments?

**DOI:** 10.3390/children10101705

**Published:** 2023-10-19

**Authors:** İrem Bağ, Seçil Çalışkan, Alaz Oya Erenel, Kübra Nur Sevimli, Merve Candan

**Affiliations:** Department of Pediatric Dentistry, Faculty of Dentistry, Eskisehir Osmangazi University, 26040 Eskisehir, Turkey; secilcaliskan@ogu.edu.tr (S.Ç.); 523120211000@ogrenci.ogu.edu.tr (A.O.E.); kubranur.sevimli@ogu.edu.tr (K.N.S.); merve.candan@ogu.edu.tr (M.C.)

**Keywords:** general anesthesia, prognosis, re-treatment, success rate

## Abstract

Background: The utilization of evidence-based approaches is crucial to achieving long-term positive outcomes for treatment performed chairside or under general anesthesia. The study aimed to evaluate if treatment modality (at the chairside or under general anesthesia) affects prognosis and the need for re-treatment. Methods: Oral-hygiene, gingival, and plaque indexes were recorded during the control appointment. The success of all treatments was evaluated according to the scoring of particular evaluation criteria. Results: A total of 1066 dental procedures were performed on 92 children. Plaque index scores were higher for patients treated under general anesthesia. The success rate of restorative procedures was 82.5% under general anesthesia and 80.6% at the chairside. There was no statistically significant difference between the mean number of restorative treatments and the need for re-treatment between general anesthesia or chairside (*p* = 0.649, *p* = 0.311). The mean number of unsuccessful endodontic treatments performed under general anesthesia was higher than performed chairside. Only two out of thirty stainless-steel crowns were decemented, all performed under general anesthesia. Conclusions: The high volume of restoration failure due to secondary caries has highlighted the need for alternative approaches to caries management, especially given the risks associated with repeat general anesthetic.

## 1. Introduction

Dental caries, one of the most common chronic diseases in the world, is a multifactorial, irreversible, and infectious disease [1,2]. Early childhood caries (ECC), formerly known as nursing bottle caries, is defined as “the presence of one or more caries (non-cavitated or cavitated lesions), missing (due to caries) or filled tooth surfaces in any primary tooth” in a child under 6 years of age. Early childhood caries remains a primary chronic disease and a public health problem [3]. If left untreated, dental caries can influence the child’s development, leading to issues such as infection, sleep disturbance, behavioral changes, appetite loss, and weight loss, and which may ultimately, result in physical, psychological, and social problems [4,5].

Most children with extensive dental caries experience difficulties in cooperation due to the long duration of treatment sessions and the fact that the procedures are completed in multiple sessions [6]. Such children may be effectively managed using basic behavior guidance techniques. However, behavioral problems that require more advanced techniques may occasionally be exhibited [7]. The American Academy of Pediatric Dentistry (AAPD) recommends that the dental treatment of children under such circumstances should be performed under general anesthesia because the treatment process can be complex and challenging [8].

The use of general anesthesia eliminates maladaptive behaviors during treatment, such as crying and resistance to procedures. Treatment under general anesthesia also ensures comprehensive and high-quality treatment in a safe environment, reduces anxiety during follow-up appointments, improves cooperation, allows for maximum contamination control in cases of restorative treatment, and provides optimal conditions such as the immobilization of the patient and the elimination of reflexes [9,10]. Such procedures may be carried out with minimal discomfort of the patient, resulting in lower levels of physical and mental stress for the dentist [9]. Children who undergo such procedures may subsequently receive rewards from their families and experience happiness due to resolving their dental issues. However, children who undergo dental extractions under anesthesia may also experience physical effects such as nausea, bleeding and fatigue, as well as psychological outcomes including hunger, fear, and anxiety [11,12,13].

When planning dental procedures (restorative treatments, endodontic treatments, preventive treatments, tooth extractions, and minor surgical procedures), both currently experienced problems and potential future issues should be considered. Although more radical approaches may be adopted under general anesthesia, compared with treatments that can be performed at the chairside, patients in such cases may subsequently require re-treatment due to their high caries risk [8,14,15,16]. In pediatric patients, poor daily oral-hygiene habits are closely associated with a need for re-treatment under general anesthesia [17].

Many factors affect the success of dental treatment in pediatric patients; they include patient age, current dental condition, behavioral/cooperative status, oral-hygiene and dietary habits, the attitude and support of the patient’s family, and post-treatment follow-ups [18,19,20]. It is possible to treat ECC both under general anesthesia or at the chairside. However, some patients cannot be treated at the chairside due to the presence of a gag reflex, the inability to adequately isolate for moisture control or a requirement for long-term comprehensive surgical procedures. In such cases, treatment under general anesthesia may be appropriate. However, procedures performed under general anesthesia may be negatively affected by factors such as the lack of a definitive diagnosis, which can influence the proposed treatment options, inadequate preoperative symptomatic evaluation, and a lack of bite-adjustment during treatment [21,22].

A review of the literature reveals that, although many common and uncommon factors are known to cause the failure of dental procedures performed under general anesthesia or at the chairside, there have been almost no comparative studies to date. This study aimed to evaluate the outcome of dental treatment under general anesthesia and at the chairside and the necessity of re-treatments for each modality. We compared our results with the prediction of hypothesis H0 that there would be no statistically significant difference between the re-treatment needs of pediatric patients treated under general anesthesia and those treated at the chairside.

## 2. Materials and Methods

The ethical approval of the present study was obtained from the Eskişehir Osmangazi University Non-Interventional Clinical Research Ethics Committee (decision dated 15 August 2023 and numbered 26). The study was conducted on patients with dental treatments performed under general anesthesia or at the chairside.

Inclusion criteria were as follows: (a) patients aged 3–14 years, both male and female; (b) patients who underwent dental treatment under general anesthesia or at the chairside; (c) patients whose treatments were carried out by a pediatric dentist (İ.B., S.Ç., M.C.) with at least 5 years of experience; (d) patients whose dental treatments were completed at least 6 months ago; (e) patients with post-operative radiographs taken within one week post-treatment; (f) patients who had procedures (endodontic/restorative treatment) with radiographically adequate treatment quality (filling without instrument separation, perforation, gross overextension or under/overfilling, and appropriate marginal integrity of the coronal restoration) [23]; and (g) patients whose parents gave informed consent for their children’s data to be used in scientific studies.

**Figure 1:**
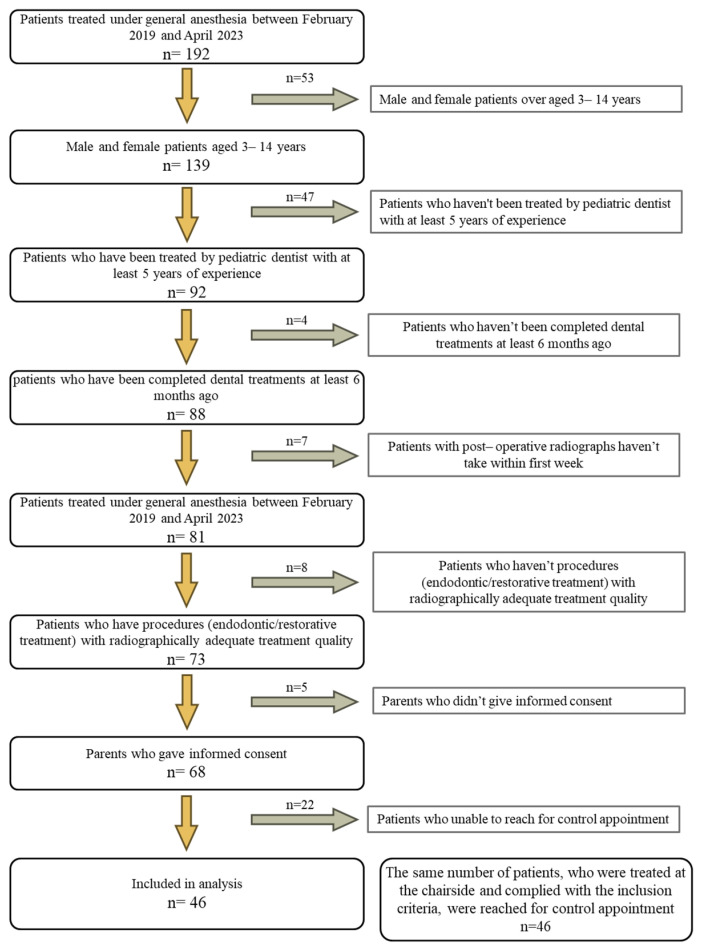
The flowchart illustrating the stages of patient selection.

The data of the 192 patients treated under general anesthesia between February 2019 and April 2023 were evaluated. In total, 68 patients who complied with the inclusion criteria for the study and whose parents gave informed consent were identified and contacted for control appointment (a session in which patients are identified according to inclusion criteria to evaluate treatment success and the need for re-treatment). Among the patients treated under general anesthesia, the parents of 46 agreed to participate in the study. The same number of patients (n = 46) treated at the chairside and in compliance with the inclusion criteria was reached for control appointment. The flowchart illustrating the stages of patient selection is presented in Figure 1. In total, 92 patients included in the study were evaluated regarding demographic characteristics (age, gender, systemic disease), post-operative cooperation, and treatment status. The Frankl behavior scale was used to record the patients’ level of cooperation. The Frankl scale consists of 4 behavior categories: (1) definitely negative; (2) negative; (3) positive; and (4) definitely positive [24,25,26]. Also, the time from treatment to control appointment was recorded.

Research data were obtained by two researchers (A.O.E. and K.N.S.). In order to standardize the evaluations of the two researchers, both individually and collectively, the Kappa test was applied to the scores of 10 patients who were not included in the sample group. As a result of this test, Kappa coefficients were determined as 0.86, 0.81, and 0.90.

The Greene and Vermillion Oral Hygiene Index, the Löe and Silness Gingival Index, and the Plaque Index were recorded during patient control appointment [27,28,29]. The success of restorative (compomer, composite, stainless steel crown), endodontic (pulpotomy, and root canal treatment), and preventive (fissure sealant, topical fluoride, and space-maintainer application) treatments was evaluated according to scoring with particular evaluation criteria. Restorations (compomer, composite, preventive resin restorations) were evaluated for retention, color match, marginal discoloration, marginal adaptation, secondary caries, surface texture, anatomic form, and postoperative sensitivity; evaluations were recorded with A, B, and C codes using modified United States Public Health Service (USPHS) criteria [30,31]. According to the codes, code A (Alpha) represents the ideal clinical situation, and B (Beta) indicates that the restoration does not need to be renewed and is clinically acceptable; however, code C (Charlie) indicates that the restoration should be renewed. Stainless-steel crowns were evaluated for clinical criteria such as crown retention, surface abrasion of the antagonist’s teeth, and the positions of the crowns regarding the gingival margin, occlusion, condition of the antagonistic tooth, location on the arch curve, and proximal contacts. Radiographic criteria such as crown margins and pulpal treatment were also assessed [32,33,34]. Endodontic treatments were evaluated for peri-radicular radiolucency, internal/external root resorption, widening of the periodontal ligament, canal obstruction, spontaneous pain, percussion/palpation pain, fistula, tooth mobility, abscess formation, and exfoliation of the treated tooth [35,36]. Fissure sealants were scored from 1 to 5 according to retention [37]. Any objections by the parents of the patients to topical fluoride application was noted, and records were kept of any such applications at any stage of treatment. The number of tooth extractions performed was also recorded. In addition, the need for space-maintainers was assessed; a record was kept when these were applied to those who needed them and also when there was any loss of space. Finally, any new caries formation was also assessed.

**Figure A1:**
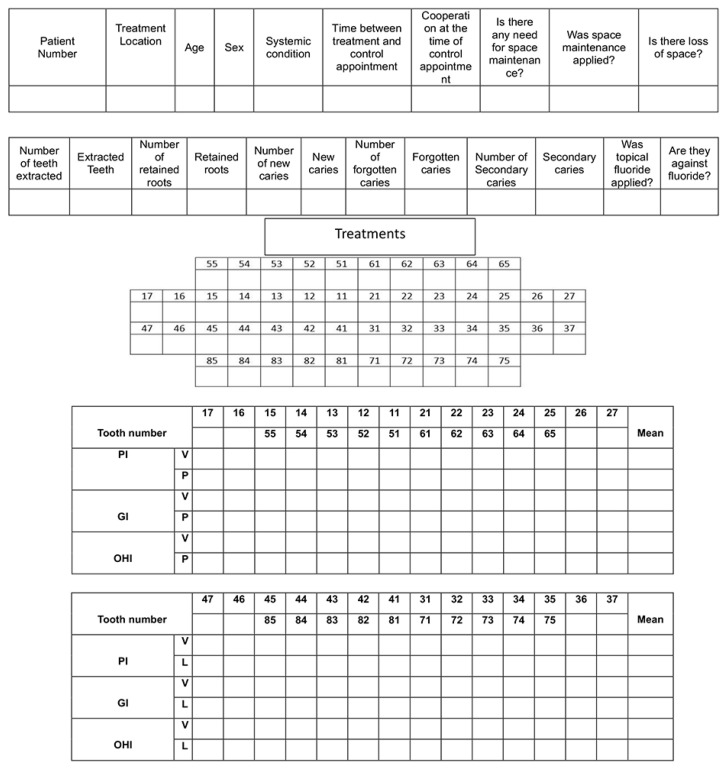

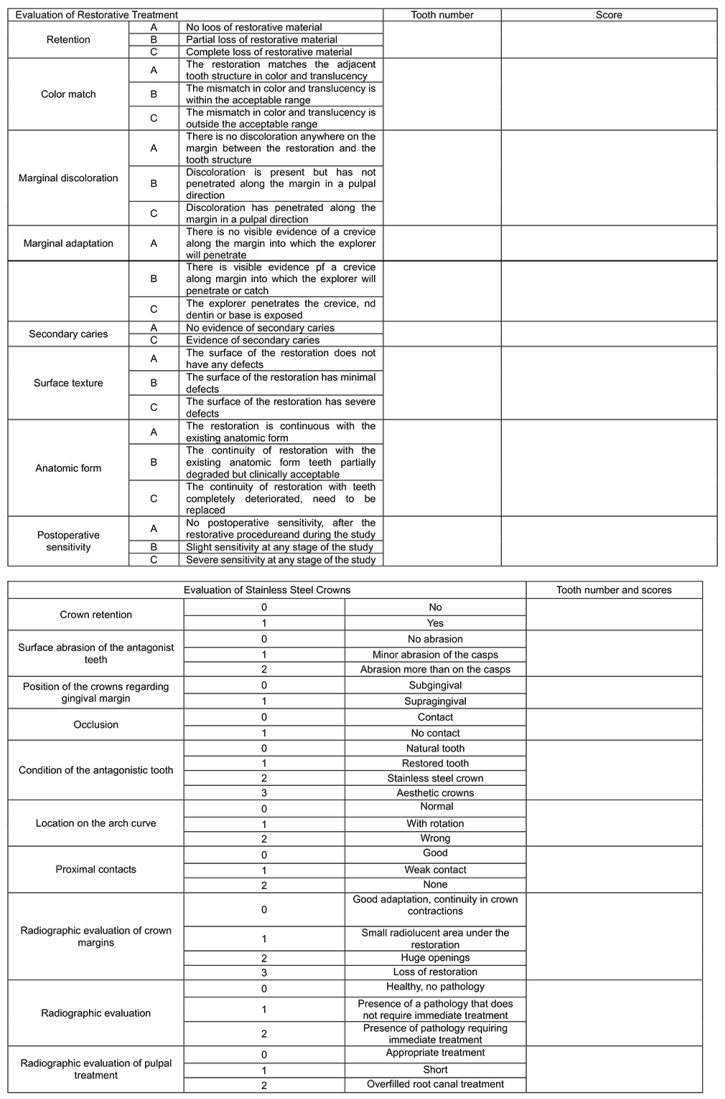

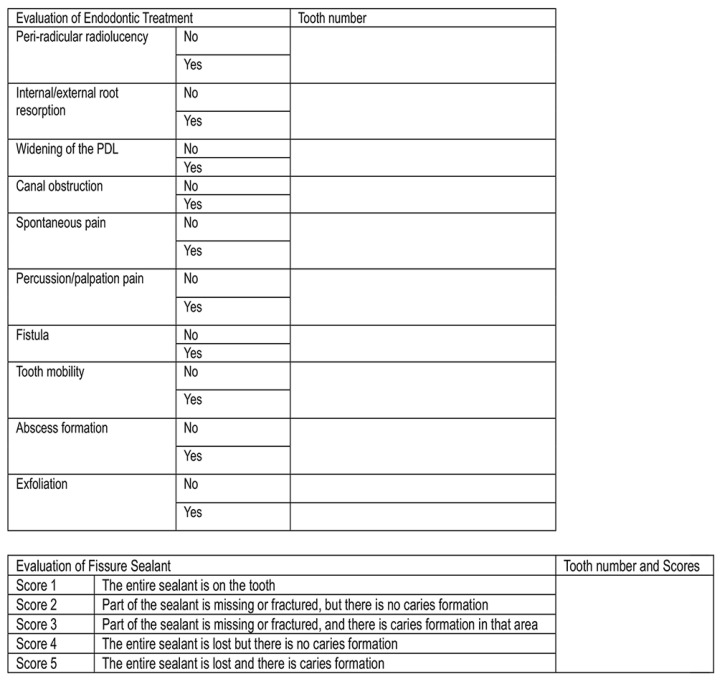
Data collection form.

The data obtained were analyzed separately for each patient according to the criteria on the data collection form (Figure A1). The success rates of dental treatments performed under general anesthesia and at the chairside were then compared and recorded.

Standard descriptive statistics (mean, standard deviation, percentages, minimum/maximum values) were used to summarize the data of all patients. The chi-square test was used to assess the distribution of categorical variables according to procedure location. The independent sample t test and one-way Analysis of Variance (ANOVA)/post-hoc Tukey tests were used to assess the difference in the mean number of total treatments and the need for re-treatments according to variables. The Pearson correlation was used to examine the relationship between the time from treatment to control appointment and the mean number of total treatments and need for re-treatments. All statistical analyses were performed using the Statistical Package for the Social Science 2.0 (IBM SPSS 22.0, Armonk, NY, USA). The statistical significance level was accepted as *p* < 0.05.

## 3. Results

In the present study, a total of 1066 dental procedures performed on 92 children were evaluated. Among the study patients included in the study, 55.4% were female, 44.6% were male, and the mean age was 8.02 ± 2.34 years. The mean time from treatment to control appointment was 13.82 ± 4.89 months for general anesthesia patients and 9.09 ± 4.01 months for chairside patients (mean: 12.51 ± 8.53 months, *p* < 0.01).

The majority (78.3%) of patients who were treated under general anesthesia were children with systemic diseases (*p* < 0.01). No statistically significant difference was found between the cooperation levels of general anesthesia and chairside patients when these were evaluated at the post-treatment control appointment (*p* = 0.052). Similarly, when oral hygiene and gingival index scores were evaluated, no statistically significant difference was observed (*p* = 0.169, *p* = 0.314). However, the plaque index scores were found to be higher in patients treated under general anesthesia (*p* = 0.038).

A comparison of the socio-demographic characteristics, systemic disease, and cooperation status of the patients enrolled in the study is shown in Table 1.

The distribution of the total number of treatments and the need for re-treatment is shown in Table 2. Of the 1066 procedures performed (831 primary teeth/77.95%, 235 permanent teeth/22.05%), 251 were unsuccessful. The overall success rate was therefore 76.45% (general anesthesia: 79.6%, at the chairside: 72.4%).

A total of 27 patients received a total of 76 fissure sealants (all resin-based); of these, 13 failed (17.10%) due to partial/total loss or caries. There was no statistically significant difference between the mean number of fissure sealants and the need for re-treatment between the general anesthesia and chairside patients (*p* = 0.222, *p* = 0.370).

In the evaluation of restorative treatment, out of the 559 restorative procedures (437 deciduous teeth/78.17%, 122 permanent teeth/21.83%) performed on 90 patients, it was determined that a total of 103 (82 primary teeth/79.6%, 21 permanent teeth/20.4%) of them required re-treatment, i.e., 73 patients. The success rate of restorative procedures was 82.5% under general anesthesia and 80.6% at the chairside. There was no statistically significant difference between the mean number of restorative treatments and need for re-treatment between the general anesthesia and chairside patients (*p* = 0.649, *p* = 0.311). Both under general anesthesia and at the chairside, the mean number of restorations requiring re-treatment was significantly higher when the time from the procedure to the control appointment was one year or longer (*p* = 0.011). There was a moderate positive correlation between the time from the procedure to the control appointment and the number of restorations requiring re-treatment (*p* = 0.036). In clinical evaluations of failures, secondary caries formation was the most common adverse finding, followed by the complete loss of restorative material (17). Secondary caries was diagnosed in 95 teeth at the control appointment. When the distribution of teeth with secondary caries formation was examined, the majority of teeth were primary (general anesthesia: 88.0%, chairside: 66.7%) and posterior teeth in the general anesthesia and chairside groups (*p* < 0.01, *p* < 0.01).

In 42 patients who underwent endodontic treatment, 101 endodontic treatments (79 pulpotomies/1 permanent tooth with mature apex, 22 root canal treatments/4 permanent teeth with mature apex) were evaluated. In total, 16 (13 general anesthesia, 3 chairside) primary tooth pulpotomies and 2 primary tooth pulpectomies (general anesthesia) were recorded as failures. The mean number of total endodontic treatments performed under general anesthesia was statistically significantly higher than that at the chairside (*p* = 0.013). The mean number of unsuccessful endodontic treatments performed under general anesthesia was higher than that at the chairside, but there was no statistically significant difference (*p* = 0.453).

Regarding clinical evaluations in terms of failure: external resorption (11), periapical radiolucency (9), internal resorption (7), widening of the periodontal ligament (6), and mobility (2) were detected in endodontic treatments performed under general anesthesia; in endodontic treatments performed at the chairside, instances of widening of the periodontal ligament (3), periapical radiolucency (2), and external resorption (2) were detected.

A total of 30 stainless-steel crowns were also evaluated, and it was found that 2 crowns in one patient failed due to decementation. All stainless-steel crowns were carried out under general anesthesia.

An evaluation of a total of 409 extractions performed on 83 patients revealed that the mean number of extractions (5.65 ± 3.91) was statistically significantly higher in patients who underwent the procedure under general anesthesia (*p* < 0.01) than in chairside patients (3.24 ± 2.16). In addition, the presence of retained roots was noted in five (1.22%) mandibular primary molars (four second molars, one first molar), two of which were performed at the chairside and three were treated under general anesthesia.

The number of patients who required space-maintainers and who were treated under general anesthesia (39) was statistically significantly higher than that treated at the chairside (30) (*p* = 0.030). There was no statistically significant difference in the loss of arch space after extraction between the general anesthesia and chairside groups (*p* = 0.582). In contrast, the presence of the loss of arch space in the general anesthesia and chairside (general anesthesia: 19.6%, chairside: 15.2%) groups was rare enough to constitute a statistically significant difference (*p* < 0.01, *p* < 0.01).

Although there were no caries in the initial radiographs, there was no statistically significant difference when 31 new carious teeth detected in the control appointment were evaluated according to the dental procedure environment (*p* = 0.448).

The fluoride application rate after treatments of patients under general anesthesia (26.1%) was lower than at the chairside (73.9%), which was a statistically significant difference (*p* < 0.01).

## 4. Discussion

In pedodontics practice, many patients, especially those of school age, may be successfully treated at the chairside so that patient rehabilitation is achieved. However, in cases of children with cooperation problems and/or systemic diseases, treatments may need to be performed under general anesthesia. Several standard or isolated factors are essential in successful treatments performed at the chairside or under general anesthesia. Based on the results of the present study, the H0 hypothesis, which was designed to compare the success of dental treatments and the need for re-treatment between general anesthesia and chairside patients, was rejected.

The primary objective of pedodontic practitioners is to ensure the sustainability of dental rehabilitation by providing high-quality dental services in a dental treatment environment. Many studies have reported differences between chairside and general anesthesia procedures in terms of treatment planning and protocols [38,39]. Although the application techniques, materials used, and indications of the treatments applied in general anesthesia and at the chairside should be analyzed, it is necessary to evaluate the prognosis of the existing treatments and determine the failure rates. However, in our literature review, we did not find any study that evaluated treatment success by comparing general anesthesia and chairside [40] procedures performed on pediatric patients.

When treatment success was evaluated according to the follow-up period, it was reported that patients treated at the chairside and under general anesthesia both exhibited positive correlations with the need for re-treatment, supporting this study’s results [18,41]. In a study that examined the reason for re-treatment, it was reported that children treated with more resin, less fissure sealant, and extraction were more likely to undergo dental treatment under general anesthesia within four years [42]. Similarly, in this study, increasing the number of resin restorations increased the need for re-treatment. In a split-mouth clinical trial, the utilization of the Hall Technique (HT) for sealing of caries demonstrated significantly superior results when compared to the standard restorative procedures, particularly in the restoration of interproximal caries. The long-term effectiveness of the HT was notably higher [43,44]. In line with the finding of the present study, it is recommended that stainless-steel crowns or the use of the HT should be preferred to resin restorations, especially when treating interproximal carious lesions. Additionally, a study found a high success rate when extractions were mostly used instead of treatments. However, in this case, it should be considered that an increase in the number of extractions may be a factor that increases dental fear.

It is essential to maintain adequate oral hygiene to ensure a favorable long-term prognosis, even when treatment is performed under ideal conditions and with appropriate standards [45,46]. Most patients undergoing procedures under general anesthesia are children with special needs or systemic diseases. Physical disabilities and parental prioritization of the existing medical condition may lead to the neglect of oral hygiene. Plaque index scores are expected to be higher in patients treated under general anesthesia [47].

In children at high risk of caries, anatomical pits and fissures on the occlusal surfaces of teeth increase the risk of carious lesions. The effective sealing of surfaces with fissure sealants can prevent caries lesions and provide a comprehensive approach to caries management [48]. It has been reported that the success rate of fissure sealants applied under general anesthesia decreases over time and ranges from 89% to 96% [20,49]. Similarly, clinical studies have shown that the retention rate of pit and fissure sealants is 82–88.6% at one year and 74% at 2.8 years [50,51]. In one clinical study in which the retention rate of fissure sealants decreased to 63% at three years, it was reported that the reason for failure was the high rate of formation of new caries lesions on adjacent-tooth surfaces [10]. Similarly, in our study, the partial or total loss of fissure sealant and failure due to caries were noted regardless of the treatment environment.

When failed treatment procedures were analyzed, restorative treatments were found to be the majority in the general anesthesia and chairside patients, in line with the findings of many similar studies. Previous studies have reported that restorative treatment procedures performed at the chairside and under general anesthesia have a failure rate of 6–41% [41,52,53,54]. In this study, restorative treatments were found to be successful in more than 80% of both general anesthesia and chairside patients, in line with success rates reported in the literature. Differences in the characteristics of the study populations, the approach of individual dentists, sample sizes, tooth types, dental materials used, cavity designs, the number of filling surfaces, and failure criteria are all important factors that may account for the discrepancies between reported results [41,52,53,54,55]. A systematic review of 31 articles on the major causes of failure of various restorations in pediatric patients highlighted the high rate of restoration failure due to secondary caries [55]. In our study, although the finding indicating the presence of secondary caries as a cause of restoration failure is consistent with the literature [55], our finding that secondary caries is more common in the posterior region is inconsistent with the literature [41,56]. In this study, resin restorations were mostly preferred over SSC for teeth with multi-face caries lesions in the posterior region. This may explain the contradictory finding of a high rate of secondary caries in the present study.

In pediatric patients, endodontic treatment should be preferred within the appropriate indication to keep their teeth functionally in the mouth and to ensure that chewing function is maintained during the transition to permanent dentition. However, it is challenging to carry out endodontic treatment in pediatric patients at the chairside due to their limited communication skills, as well as difficulties in cooperation and isolation [57]. The advantages of endodontic treatment under general anesthesia include aseptic working conditions, the absence of any need for pain or anxiety management, and increased opening of the mouth. One published guideline on treatment under general anesthesia recommends that comprehensive or conservative treatment be chosen instead of extraction [58]. In line with this recommendation, in the present study, the number of endodontic treatments performed under general anesthesia was higher, and pulpotomies were primarily performed on primary teeth. Different failure rates for pulpotomy and pulpectomy have been reported in the literature [49,59,60,61,62]. In this study, in which pulpotomy mainly was performed as an endodontic treatment, a high treatment success rate was observed in the follow-up periods ranging from 7 to 24 months, but in contrast to the literature, the failure rate of the pulpotomy treatment of primary teeth performed under general anesthesia was high [60,63,64,65]. In the present study, the overall success rate of endodontic treatment was 83%; however, the number of failed endodontic treatments performed under general anesthesia was statistically significantly higher than the corresponding number for at the chairside.

Although it has been emphasized that endodontic treatment under general anesthesia can be performed under almost ideal conditions, it should also be noted that the bite adjustments of patients after restoration cannot be evaluated multilaterally with fixed reference points and that the distribution of forces transmitted to the apex may be related to the preparation of the ground for pathology. Although this applies to any restorative procedure performed under general anesthesia, it should not be overlooked that a decrease in tissue tolerance due to a lack of reparative response may be a disadvantage, especially in endodontically treated primary teeth. When the results of this study were analyzed in terms of mode of failure, it was found that radiographic pathologies were mainly present in the absence of clinical symptoms. Although the clinical success of formocresol used in pulpotomy (which accounts for the majority of failed endodontic treatments) is high, many studies have reported low radiographic success due to pathologies such as external root resorption, periapical radiolucency, and internal resorption [66,67,68].

The use of stainless-steel crowns in dental procedures, especially in the case of endodontically treated teeth, results in more successful long-term outcomes, compared with other restoration methods, due to their durability and sealing properties. The high success rate of SSCs fitted under general anesthesia in the present study is consistent with that in the literature [44,69]. While resin restorations are covered by health insurance in the country of the present study, the application of SCCs is often associated with an additional cost, which is thought to negatively affect the acceptance rate of parents at the chairside. The results of similar studies [70,71,72] suggest that the attitude of parents towards the additional cost is more favorable in treatments performed under general anesthesia, as it may create the need for re-admission to general anesthesia for the rehabilitation of restoration losses.

Although the success of treatment in pediatric dentistry depends on many factors, including the practitioner and the dental materials and techniques used, a positive attitude of parents and children toward oral-hygiene requirements is essential to ensure a favorable long-term prognosis. In line with the results of this study, it has been reported that even when children with early childhood caries have their condition fully treated, new caries lesions can form shortly after treatment. In one study evaluating the rate of new caries lesions, 8.5% of the patients developed new caries lesions within 6 months, and 18.8% developed new caries lesions within 12 months of follow-up periods [20].

The high number of new and secondary caries detected in our study demonstrates the need for regular preventive treatments during follow-ups. Considering that dental treatment under general anesthesia results in an increased risk of morbidity and mortality, as well as a higher cost of treatment, it is of great importance that preventive dentistry practices should be included in the planning of treatment, not only for existing problems, but also for those that may occur, so that patients at high risk of caries do not need to be treated again [73]. However, the results of the present study showed that many parents of patients treated under general anesthesia do not allow fluoride application, even though it is a non-invasive treatment.

The limitations of this study include the lack of a single practitioner performing the procedures, differences in restorative materials, and no examination of cavity dimensions. Although the aim of this study was to evaluate whether the choice of treatment modality (at chairside or under general anesthesia) affects prognosis and the need for re-treatment, the effect of sedation, which represents another treatment modality, was not evaluated. This limitation is due to the faculty’s inability to provide sedation facilities. It is important to note that, typically, radiological assessments should be conducted immediately. However, in this study, these assessments were performed one week later due to the requirement for specialized technical equipment located within the general anesthesia environment. No study has been published in the current literature to evaluate the prognosis of treatments and the need for re-treatment under general anesthesia and at the chairside. The results obtained within the limitations will guide future research. Through this comprehensive study, we have taken significant strides toward filling a critical gap in dental research, and it is anticipated that the success of both general anesthesia and chairside treatments may now be substantially enhanced, with the potential to prevent the need for re-treatment given appropriate conditions such as clinical follow-up and sustained oral hygiene. There is a need for studies with long follow-up periods in which different dental treatment materials and techniques are comparatively evaluated on a large number of patients. Additionally, conducting studies in which all modalities (general anesthesia, sedation, and chairside) are evaluated comparatively will contribute to the literature.

## 5. Conclusions

The need for re-treatment increases with the formation of new caries due to poor oral-hygiene habits. The low oral hygiene index scores of patients treated under general anesthesia indicate poor oral hygiene practices. In both modalities, the cause of restoration failure is mostly secondary caries. Considering the risks associated with repeat general anesthesia, the failure of restorations under the ideal conditions of general anesthetic represents an important problem that requires innovative solutions. For this reason, it has become clear that restorative options with a high success rate in the long term should be preferred.

## Figures and Tables

**Table 1:** Distribution of the socio-demographic characteristics of patients included in the study in the general anesthesia and chairside groups.

		General Anesthesia n (%)	Chairside n (%)	Total	*p*
Sex	Female	23 (50)	28 (60.9)	51	0.249
	Male	23 (50)	18 (39.1)	41	
Age	0–6	20 (43.5)	0	20	<0.01 *
	6–9	23 (50)	15 (32.6)	38	
	9–12	3 (6.5)	31 (67.4)	34	
Systemic Disease	Yes	36 (78.3)	45 (97.8)	81	<0.01 *
	No	10 (21.7)	1 (2.2)	11	
Cooperation	Score 1	1 (2.2)	0	1	0.052
	Score 2	7 (15.2)	2 (4.3)	9	
	Score 3	16 (34.8)	10 (21.7)	26	
	Score 4	22 (47.8)	34 (73.9)	56	

* *p* < 0.05; Chi-square test.

**Table 2:** The distribution of the total number of treatments and the need for re-treatment in the general anesthesia and chairside groups.

Treatment Type		General Anesthesia (Mean ± SD)	Chairside (Mean ± SD)	*p*
Restorative	Total	6.21 ± 2.89	5.93 ± 3.04	0.649
	Re-treatment Needs	2.30 ± 2.08	2.74 ± 2.00	0.311
Endodontic	Total	2.82 ± 1.49	1.64 ± 1.15	0.013 *
	Unsuccessful	1.50 ± 1.08	1.00	0.453
Fissure Sealant	Total	0.63 ± 1.37	1.00 ± 1.50	0.222
	Re-treatment Needs	0.48 ± 1.07	0.30 ± 0.76	0.370

* *p* < 0.05; *t*-test.

## Data Availability

The data presented in this study are available on request from the corresponding author.
